# Obituary

**Published:** 1890-07

**Authors:** 


### Obituary.

Died, at the residence of his father, in New York City, of pneumonia, May 31st, Clinton Atkinson, eldest son of Dr. Wm. H. Atkinson. Dr. Atkinson had a struggle of several weeks with the disease to which he finally succumbed. He was a graduate of the College of Physicians and Surgeons, of New York, and was above the average in the science and art of medicine and dentistry. He had spent the most of his active professional life in the office of his father, and had the advantage of the superior training which his father was so abundantly able to give him.

It seems, according to human views, unfortunate that persons of such ability should be taken away in early manhood, and in life's highest activities. His father, and the family, will have the warmest sympathy of the entire profession, and the prayer of all is, and will be, that they may be sustained and comforted in this, their great trial.

NOTICE.—THE NATIONAL ASSOCIATION OF DENTAL FACULTIES will meet on Monday, August 4th, at 10 A. M., at Excelsior Springs, Mo. J. S. MARSHALL, Secy.

